# A novel liver-specific fluorescent anti-cancer drug delivery system using indocyanine green

**DOI:** 10.1038/s41598-019-39269-0

**Published:** 2019-02-28

**Authors:** Yoshinori Inagaki, Takashi Kokudo, Mako Kamiya, Shin-nosuke Uno, Masumitsu Sato, Junichi Kaneko, Norihiro Kokudo, Yasuteru Urano, Kiyoshi Hasegawa

**Affiliations:** 1 Hepato-Biliary-Pancreatic Surgery Division and Artificial Organ and Transplantation Division, Department of Surgery, Graduate School of Medicine, The University of Tokyo, Tokyo, Japan; 2 Laboratory of Chemical Biology & Molecular Imaging, Graduate School of Medicine, The University of Tokyo, Tokyo, Japan; 3 Laboratory of Chemistry and Biology, Graduate School of Pharmaceutical Sciences, The University of Tokyo, Tokyo, Japan

## Abstract

Indocyanine green (ICG) accumulates only in hepatocytes and their malignant counterpart, hepatocellular carcinoma (HCC). We have developed ICG-conjugated anti-cancer drugs and noted their significant accumulation in HCC cells both *in vitro* and *in vivo*. ICG-conjugated gemcitabine was less toxic to normal cells and it had superior anti-tumor action compared to gemcitabine alone in a subcutaneous tumor xenograft. ICG conjugation can provide a novel fluorescent drug delivery system for treatment of liver cancer and this system can be used to both diagnose and treat HCC.

## Introduction

Hepatocellular carcinoma (HCC) is the second leading cause of cancer mortality worldwide^1^. Patients often present with advanced disease at the time of diagnosis. Many cytotoxic chemotherapeutic agents, such as gemcitabine (Gem) and doxorubicin (Dox), have been used in an effort to treat advanced HCC, however, currently available evidence on their efficacy is not sufficient to change current treatment guidelines^2–4^. This is probably due to the fact that HCC patients often suffer from underlying liver disease, chronic hepatitis, or cirrhosis, and HCC patients cannot tolerate the same dose as patients with other types of cancer. In order to overcome this limitation, direct injection into the hepatic artery, known as hepatic arterial infusion chemotherapy (HAIC), has been proposed, however, evidence of the oncological benefit of HAIC is still limited^5^. To date, the only recommended systemic treatment for advanced HCC is multi-kinase inhibitors^6,7^. However, the reported survival benefit is as little as 3 months on average, so a specific drug delivery system (DDS) is still needed for treatment of HCC.

Since indocyanine green (ICG) is a fluorescent material that is exclusively taken up by hepatocytes, ICG is universally used for liver function analysis^8,9^. We have recently reported that ICG accumulates in HCC cells as well and they developed an ICG-fluorescence navigation system for detecting HCC lesions during liver resection^10,11^. Since ICG persists in a patient’s HCC tissue for longer than 2 weeks, this prolonged and specific accumulation of ICG could presumably facilitate development of a novel fluorescent DDS that can be used to both diagnose and treat HCC.

## Materials and Methods

### Cell lines and animals

HuH-7 (a well differentiated HCC cell line) cells were obtained from the Japanese Collection of Research Bioresources Cell Bank (JCRB, Osaka, Japan). HepG2 (a well-differentiated HCC cell line) cells were obtained from the European Collection of Animal Cell Cultures (ECACC, Salisbury, UK). HCT116 (colorectal carcinoma cell line) cells were obtained from the American Type Culture Collection (ATCC, Rockville, MD, USA). Cells were maintained in Dulbecco’s modified Eagle’s Medium (Invitrogen, Carlsbad, CA, USA) with 10% fetal bovine serum (Invitrogen, Carlsbad) at 37 °C in a humid atmosphere (5% CO_2_–95% air) and harvested by brief incubation in an Enzyme-free Cell Dissociation Solution (Millipore Co., Bedford, MA, USA). Human umbilical vein endothelial cells (HUVEC) were maintained in EGM-2 medium (Lonza Japan, Tokyo, Japan).

Male BALB/c nude mice were purchased from Charles River Laboratories Japan, Inc. (Yokohama, Japan), and used at 5–6 weeks of age. Animals were kept under specific pathogen-free conditions. All procedures of animal experiment were performed in accordance with protocols approved by the Institutional Committee for Animal Research of the University of Tokyo.

### Synthesis of ICG-conjugated gemcitabine

ICG-CO_2_H, an ICG derivative, was purchased from Goryo Chemical, Inc. (Tokyo, Japan). 1-Hydroxybenzotriazole (HOBt) (4.6 mg, 0.034 mmol, 2 eq), 1-ethyl-3-(3-dimethylaminopropyl)carbodiimide hydrochloride (WSCD) (6.5 mg, 0.034 mmol, 2 eq), and N,N-diisopropylethylamine (14.8 μL, 0.085 mmol, 5 eq) were added to a solution of 3′,5′-O-bis(tert-butoxycarbonyl) gemcitabine (11.8 mg, 0.025 mmol, 1.5 eq)^12^ and ICG-CO_2_H (12.4 mg, 0.017 mmol, 1 eq) in dry DMF (2 mL). The reaction mixture was stirred under an argon atmosphere at r.t. for 26 h, and then the solvent was evaporated. The residue was dissolved in TFA (2 mL) and the solution was stirred at r.t. for 5 h. The solvent was evaporated and the residue was purified by preparative HPLC using eluent A (H_2_O with 0.1% TFA and 1% CH_3_CN) and eluent B (CH_3_CN with 1% H_2_O) (A/B = 90/10 to 0/100 for 40 min) to yield ICG-Gem (5.6 mg, 34% for 2 steps).

^1 ^H NMR (400 MHz, MeOD): *δ* 8.32 (d, *J* = 7.6 Hz, 1 H), 8.21 (t, *J* = 7.3 Hz, 2 H), 8.01–7.94 (m, 6 H), 7.64–7.59 (m, 4 H), 7.54 (d, *J* = 8.9 Hz, 1 H), 7.46 (q, *J* = 7.2 Hz, 2 H), 7.39 (d, *J* = 7.6 Hz, 1 H), 6.61–6.53 (m, 2 H), 6.41 (d, *J* = 13.4 Hz, 1 H), 6.30–6.22 (m, 2 H), 4.34–4.20 (m, 5 H), 4.00–5.96 (m, 2 H), 3.84–3.81 (m, 1 H), 2.93 (t, *J* = 6.8 Hz, 2 H), 2.49 (t, *J* = 7.0 Hz, 2 H), 2.05–1.88 (m, 20 H), 1.78 (quin, *J* = 7.3 Hz, 2 H), 1.57–1.50 (m, 2 H), 1.33–1.29 (m, 1 H); HRMS (*m/z*): [M + Na]^+^ calcd. for C_54_H_59_F_2_N_5_NaO_8_S, 998.39446; found, 998.39318.

ICG-Gem was dissolved in anhydrous DMSO to obtain 10 mM stock solutions, and their concentration was adjusted using the molar extinction coefficient for ICG (*ε* = 1.5 × 10^5^ M^−1^cm^−1^).

### Evaluation of intracellular fluorescence *in vitro*

Continuously cultured HuH-7 cells were harvested in tubes and resuspended in conditional medium containing 10% FBS after they were washed with PBS. The cells were seeded in a 4-well chamber slide at a density of 1 × 10^5^ cells in 1000 μL, incubated with ICG-Gem and ICG-Dox for 24 h, and then additionally incubated for 24 h after the ICG conjugates were removed. Fluorescence of ICG conjugates in cultured cells was detected using a fluorescence microscope (Hamamatsu Photonics, Hamamatsu, Japan).

### Cell toxicity assay *in vitro*

Continuously cultured HuH-7, HepG2 and HUVEC cells were harvested in tubes and resuspended in conditional medium containing 10% FBS after they were washed with PBS. The cells were seeded in triplicate in 96-well plates at a density of 5 × 10^3^ cells in 200 μL and incubated with a series of concentrations of ICG-Gem for 48 h at 37 °C in a 5% CO_2_ atmosphere. Cell viability was evaluated using a methylthiazole tetrazolium (MTT) cell proliferation assay. Briefly, thiazolyl blue tetrazolium bromide (Sigma-Aldrich Japan, Tokyo, Japan) was added and incubation was continued for 4 h. Then, cells were solubilized, and absorbance at 550 nm was measured.

### Tumor growth assay *in vivo*

Continuously cultured HuH-7 cells were harvested in tubes and resuspended in serum-free medium after they were washed with PBS. Each mouse was subcutaneously injected in the abdomen with 5 × 10^6^ HuH-7 cells in 200 μL of serum-free medium containing 50% Matrigel (Becton-Dickinson, Franklin Lakes NJ, USA). Point-zero-four mg/kg of ICG-Gem was injected intravenously via a tail vein 10 d after transplantation and the distribution of ICG-derived fluorescence was observed using a fluorescence imaging system (Hamamatsu Photonics). A model of HAIC was created by directly injecting 0.2 mg/kg of ICG-Gem into an HuH-7 tumor xenograft 10 d after transplantation. HuH-7 tumor size was measured every 3 d, and tumor volume was monitored for a total of 9 d following initial treatment. Tumor volume (mm^3^) was calculated using the formula a x b x c (mm), where a, b, and c are the width, depth, and height of the tumor, respectively.

### Statistical analysis

Statistical analysis was performed with the Student’s t-test using the software SPSS 11.5. P < 0.05 was considered to indicate a significant difference. All experiments were performed in triplicate.

## Results and Discussion

### Development of an ICG-conjugated anti-cancer drug

**Figure 1:**
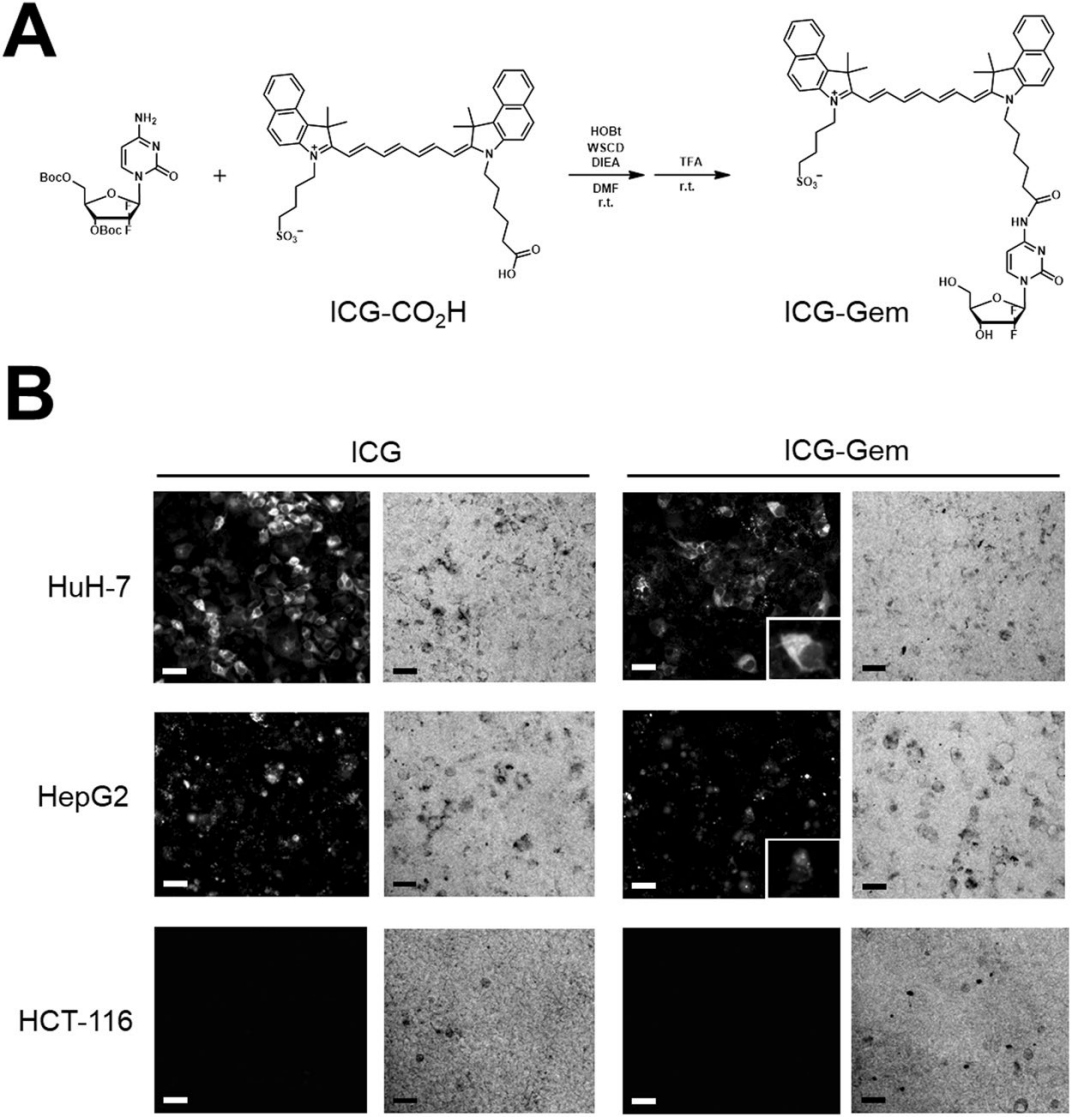
Chemical structure and fluorescent distribution of an indocyanine green (ICG)-conjugated anti-cancer drug. (**A**) ICG is conjugated with gemcitabine (Gem) through coupling reactions, followed by deprotection. (**B**) Fluorescent and bright image (x100) of hepatocellular carcinoma (HCC) cells and colon cancer cell (HCT116), 24 h after exposure to ICG or ICG-Gem. Scale bar = 50 μm. The right lower panel shows the magnified fluorescent image of HCC cells 24 h after exposure to ICG-Gem.

Since a study has already described ICG-conjugated antibodies that use the amine group as a conjugation site^13^, we initially attempted to use Dox and Gem as potential anti-cancer drugs for ICG conjugation since they possess an amine group and have already been used to treat HCC^2,3^. A conjugate of ICG and Gem was synthesized by coupling ICG-CO_2_H, an ICG derivative, and Boc-protected Gem, followed by deprotection (Fig. 1A). ICG-Dox was synthesized as well using its amine group. After HCC cells were incubated for 24 h, a fluorescence microscope revealed ICG-Gem accumulation in the cytosol of both HCC cell lines, HuH-7 and HepG2, and this accumulation was not noted in colon cancer cell (HCT116) (Fig. 1B). ICG fluorescence was also detected in the nuclei of HCC cells (Fig. 1B right lower panel) although it was weaker than that in cytosol. Similar accumulation was observed concerning ICG-Dox (Supplementary Fig. 1A). These results indicate that ICG can be used as a DDS carrier to ferry an anti-cancer drug so that it accumulates in HCC cells.

### Effect of the ICG-conjugated anti-cancer drug *in vitro*

**Figure 2:**
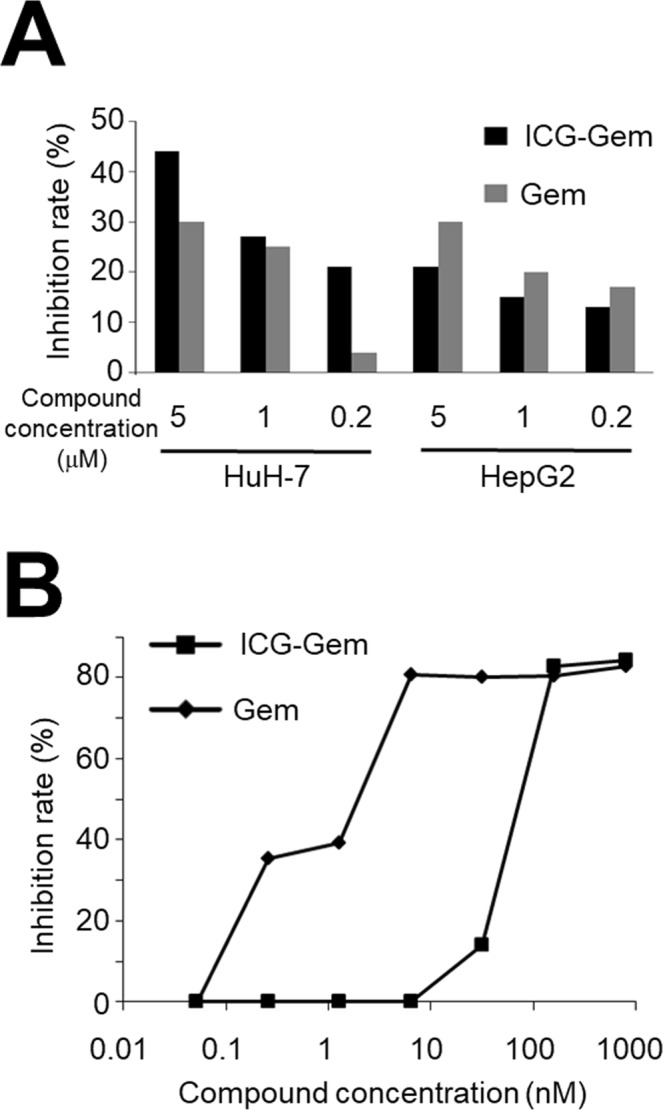
Effect of an indocyanine green (ICG)-conjugated anti-cancer drug *in vitro*. (**A**) A methylthiazole tetrazolium (MTT) cell proliferation assay of HCC cells 24 h after exposure to ICG-Gem or Gem. (**B**) An MTT assay of human umbilical vein endothelial cells (HUVEC) 24 h after exposure to ICG-Gem or Gem.

The anti-cancer action of these ICG-conjugated anti-cancer drugs was evaluated. MTT cell proliferation assay of HCC cells was performed. Although ICG-Gem and Gem had similar anti-cancer action against HepG2 cells *in vitro*, ICG-Gem demonstrated a superior cytotoxic effect in HuH-7 cells at lower concentration (Fig. 2A). This result is consistent with the results that ICG-Gem better accumulates in HuH-7 cells than in HepG2 cells (Fig. 1B). However, the cytotoxic effect was significantly impaired concerning ICG-Dox (Supplementary Fig. 1B). ICG-Gem was less toxic to HUVEC than Gem (Fig. 2B).

### Effect of the ICG-gemcitabine complex *in vivo*

**Figure 3:**
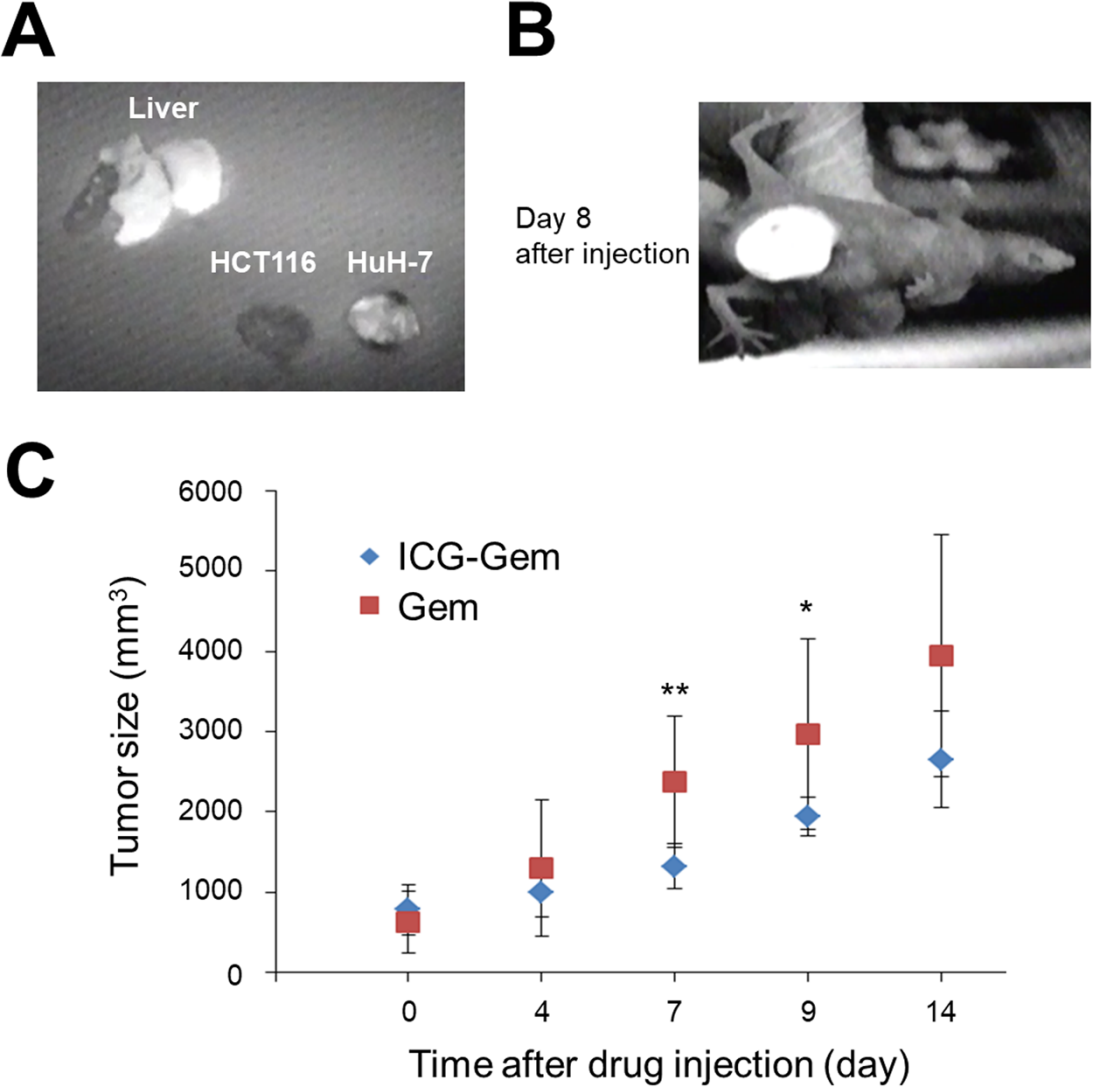
Effect of an indocyanine green (ICG)-conjugated anti-cancer drug *in vivo*. (**A**) Distribution of ICG-Gem *in vivo* 24 h after intravenous administration. (**B**) Fluorescent image of the subcutaneous tumor 8 d after direct injection of ICG-Gem into the tumor. (**C**) Time course of the subcutaneous tumor volume after direct injection of ICG-Gem or Gem into the tumor. HCT116: colon cancer cell line, Huh7: hepatocellular carcinoma cell line. *P < 0.05, **P < 0.01.

ICG-Gem was administered via the tail vein of tumor-bearing nude mice and fluorescent distribution was analyzed 24 h after injection. ICG-Gem accumulated not in the colon cancer cell (HCT116) tumor xenograft but to HCC cell (HuH-7) tumor xenograft (Fig. 3A). Although Gem itself is reported to be excreted via the urine^14,15^, ICG-Gem accumulated solely in the normal liver and ICG-Gem was excreted via the bile ducts and stool without urinary excretion. Therefore, ICG-Gem would have difficulty serving as a systemic chemotherapy agent and more selective administration of an agent to HCC cells might be necessary. Thus, ICG-Gem was directly injected into the subcutaneous tumor xenograft as a model of HAIC. The accumulation of ICG-Gem was detected in the subcutaneous tumor xenograft after longer than 1 week (Fig. 3B). There were no obvious adverse reactions, such as appetite loss or weight loss, in the treated mice. Furthermore, the tumor volume remained significantly smaller after a single injection of ICG-Gem compared to Gem (Fig. 3C).

The current results revealed that an ICG-conjugated anti-cancer drug accumulated in HCC cells both *in vitro* and *in vivo*. Results also revealed that conjugated ICG was less toxic to normal cells. After ICG-Gem was directly injected into a subcutaneous tumor xenograft, it persisted in the tumor for longer than 1 week and it had superior anti-tumor action compared to Gem alone.

We recently reported that intravenous administration of ICG results in its accumulation in around 90% of HCCs^11^. This trait could presumably be used to develop a novel DDS. Since both Dox and Gem possess an amine group, these drugs were used initially. Indeed, both ICG-Gem and ICG-Dox accumulated in the HCC cells *in vitro*; however, ICG-Gem remained cytotoxic, but ICG-Dox did not (Supplementary Fig. 1). Although ICG can be used as a carrier for an HCC-specific DDS, cytotoxicity of an ICG conjugate depends on the structure of the conjugated component.

Since slight ICG fluorescence was detected in nuclei after ICG-Gem administration (Fig. 1B), ICG-Gem may act in a manner like Gem in the nucleus. ICG-Gem and Gem had similar toxicity *in vitro*, however ICG-Gem was superior to Gem *in vivo*. These results indicate that ICG conjugation can provide a novel fluorescent DDS and that this DDS can be used for both diagnosis and treatment of HCC. The mechanism of anti-cancer action of these ICG-conjugated drugs must be studied further.

Conjugation of ICG resulted in ICG-Gem and ICG-Dox accumulating in the cytosol of HCC cells. In addition, ICG-Gem was excreted via the bile ducts whereas Gem itself was excreted via the urine^14,15^. These findings indicate that ICG and ICG-conjugated anti-cancer drugs are transported inside HCC cells in a similar manner. Recently, Kagawa *et al*. reported that organic anion-transporting polypeptide 1B3 (OATP1B3) is the transporter responsible for ICG clearance^16^. Therefore, OATP1B3 might also be a transporter responsible for the action of this novel ICG-mediated DDS.

Although accumulating the character of ICG to HCC cells were sustained even after gemcitabine conjugation, the water solubility has changed significantly. Furthermore, although ICG does not accumulate in the normal liver, as shown in Supplementary Fig. 2, ICG-Gem accumulated in normal liver (Fig. 3A). These results indicate that the structure of ICG changed after gemcitabine conjugation, resulting in the different behavior between ICG and ICG-Gem. However, this issue needs further investigation with structural determination.

One of the limitations of the current study is the fact that ICG-Gem also accumulated in the normal liver. Since ICG itself does not accumulate in the normal liver, this accumulation is probably due to the structural changes in ICG caused by Gem conjugation. Accumulation in the normal liver could cause liver damage, so a protocol for more specific administration is needed to use ICG-Gem in a clinical setting. The current study focused on use of ICG-Gem in HAIC or some other locoregional therapy, and ICG-Gem was directly injected in this study. This limitation might also be overcome by identifying ICG-conjugated anti-cancer drugs that do not accumulate in the normal liver. Although the data are relatively preliminary and needs further investigation before clinical application, this is a novel drug delivery system using the fluorescent agent itself as a carrier. This agent can be applied not only for diagnostic use but also for therapeutic purposes. In addition, it may also be applied to photodynamic therapy that have been previously reported using ICG^17,18^.

In conclusion, ICG conjugation can provide a novel HCC-specific fluorescent DDS. ICG-Gem may be a promising HCC-specific agent.

## Supplementary information

Supplementary figures and legends [[Please replace with revised ESM file from the attachment]]
