# Supplementary material for: A novel liver-specific fluorescent anti-cancer drug delivery system using indocyanine green
Source: Sci Rep. 2019 Feb 28;9:3044. doi: 10.1038/s41598-019-39269-0 (PMC6395603; doi:10.1038/s41598-019-39269-0)
Supplement: Supplementary file 1 — Supplementary figures and legends [[Please replace with revised ESM file from the attachment]] [file 41598_2019_39269_MOESM1_ESM.pdf]

Supplementary information

A novel liver-specific fluorescent anti-cancer drug delivery system using indocyanine green

Yoshinori Inagaki[1,*], Takashi Kokudo[1,*], Mako Kamiya[2], Shin-nosuke Uno[2], Masumitsu Sato[1], Junichi Kaneko[1], Norihiro Kokudo[1], Yasuteru Urano[2], Kiyoshi Hasegawa[1]

[1]Hepato-Biliary-Pancreatic Surgery Division and Artificial Organ and Transplantation Division, Department of Surgery, Graduate School of Medicine, The University of Tokyo

[2]Laboratory of Chemical Biology & Molecular Imaging, Graduate School of Medicine, The University of Tokyo

* Authors share co-first authorship.

Corresponding author: Kiyoshi Hasegawa

Address: 7-3-1 Hongo, Bunkyo-ku, Tokyo 113-8655, Japan.

Tel: +81 338155411; fax: +81 356843989

E-mail address: kihase-tky@umin.ac.jp

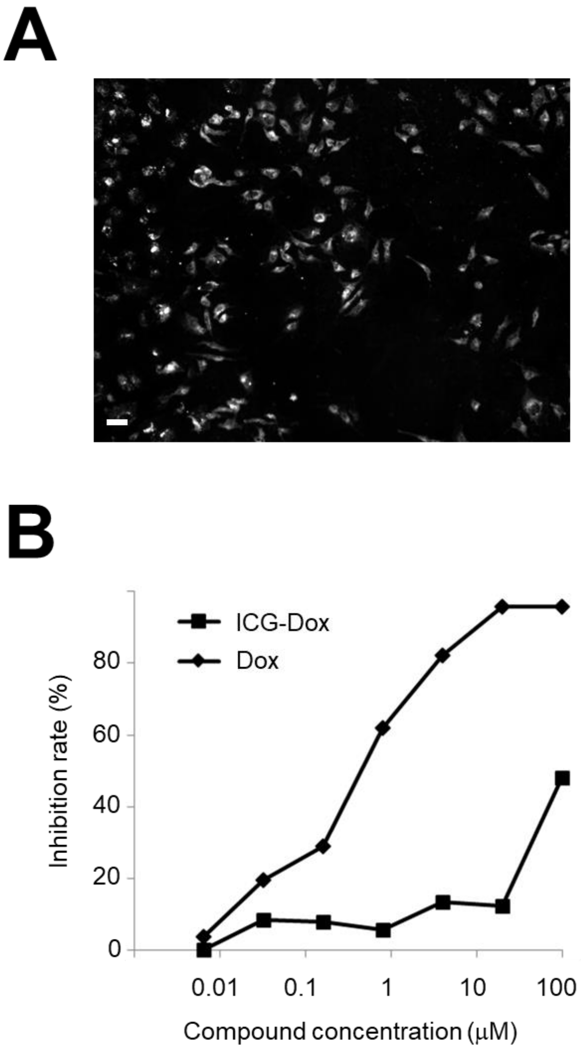

Supplementary Figure 1. Effect of an indocyanine green (ICG)-conjugated doxorubicin (ICG-Dox) *in vitro*. (A) Fluorescent image (x100) of hepatocellular carcinoma (HCC) cells 24 h after exposure to ICG-Dox. Scale bar = 50 μm. (B) An MTT assay of HuH-7 cells 24 h after exposure to ICG-Dox or Dox.

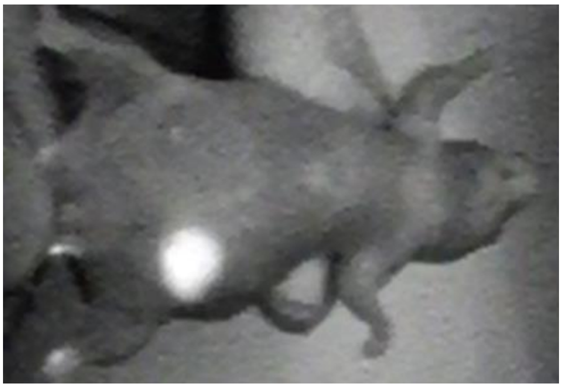

Supplementary Figure 2. Distribution of indocyanine green (ICG) *in vivo*. Fluorescent image of ICG *in vivo* 24 h after intravenous administration.
